# Barriers to, and Facilitators of, Diabetes Self-management in the Dialysis Population: A Narrative Review and Implications for Research

**DOI:** 10.1177/20543581251359734

**Published:** 2025-07-29

**Authors:** Kokab Younis, Graham McCaffrey, Kathryn King Shier, Shelley Raffin Bouchal, Robert R. Quinn

**Affiliations:** 1 Faculty of Nursing, University of Calgary, AB, Canada; 2 Department of Medicine, University of Calgary, AB, Canada; 3 Department of Community Health Sciences, University of Calgary, AB, Canada

**Keywords:** diabetes self-management, barriers, facilitators, chronic kidney disease, diabetes mellitus

## Abstract

**Purpose of review:**

Patients with both diabetes and kidney failure requiring dialysis are a complex population that is at risk of diabetes-related complications, hospitalizations, and mortality. Due to the significant illness burden, self-management of diabetes becomes challenging. The purpose of this review was to identify and synthesize the literature on barriers to, and facilitators of, diabetes self-management among patients with both diabetes and kidney failure requiring dialysis.

**Sources of information:**

We conducted a search of health care databases (CINAHL, PubMed, OVID Medline) to find studies that were focused on exploring barriers to, and facilitators of, diabetes self-management in this population. We included English-language qualitative, quantitative, and mixed-methods studies.

**Methods:**

We performed a focused narrative review assessing barriers and facilitators to diabetes management among patients with chronic kidney disease. The literature was critically analyzed using various appraisal tools, and thematic analysis was performed.

**Key findings:**

A total of 134 articles were identified. Eight articles met inclusion criteria. A review of the articles revealed barriers in diabetes self-management covering 5 themes: financial limitations, limited access to healthcare services, siloed and fragmented care, increased complexity of the dietary regimen, and the higher burden of health. Three themes were revealed pertaining to facilitators of diabetes self-management: self-management support and education, coordinated care between healthcare providers, and family support.

**Limitations:**

The literature search was in-depth and comprehensive, but not exhaustive. Also, we restricted our search criteria to articles published in the English language.

**Implications:**

There can be challenges living with multiple chronic conditions, especially for those with comorbid diabetes and kidney failure requiring dialysis. This study underscores the urgent need for quality improvement and research initiatives to support these individuals. In addition, conducting further qualitative research to explore the perspectives of dialysis patients, their health care professionals, and caregivers would be beneficial.

## Abrégé

**Justification:**

Les patients diabétiques qui souffrent également d’insuffisance rénale nécessitant une dialyse sont une population complexe qui présente un risque élevé de complications, d’hospitalisation et de mortalité lié au diabète. Cet important fardeau rend difficile l’autogestion du diabète. Cette étude avait pour objectif de répertorier et de synthétiser la documentation portant sur les facteurs limitant ou facilitant l’autogestion du diabète chez les patients diabétiques atteints d’insuffisance rénale nécessitant une dialyse.

**Sources de l’information:**

Nous avons consulté les bases de données de santé (CINAHL, PubMed, OVID Medline) à la recherche d’études explorant les obstacles à l’autogestion du diabète et les facteurs qui la facilitent dans cette population de patients. Nous avons inclus des études qualitatives, quantitatives et mixtes en langue anglaise.

**Méthodologie:**

Une revue narrative ciblée évaluant les obstacles et les facilitateurs à la prise en charge du diabète chez les patients diabétiques atteints d’insuffisance rénale chronique. La documentation a fait l’objet d’une analyse rigoureuse à l’aide de plusieurs outils d’évaluation; une analyse thématique a également été effectuée.

**Principaux résultats:**

Un total de 134 articles a été identifié, desquels huit répondaient aux critères d’inclusion. L’examen des articles retenus a révélé cinq thèmes pour les obstacles à l’autogestion du diabète: les limitations financières, l’accès limité aux services de santé, le cloisonnement et la fragmentation des soins, la complexité accrue de la diète et le fardeau élevé lié à la santé. Trois thèmes ont été dégagés pour les facilitateurs: le soutien et l’éducation concernant l’autogestion, la coordination des soins entre les prestataires de soins de santé et le soutien familial.

**Limites:**

La recherche documentaire était rigoureuse et approfondie, mais non exhaustive. Nous avons limité nos critères de recherche aux articles publiés en anglais.

**Conclusion:**

Vivre avec plusieurs maladies chroniques peut représenter un défi, en particulier pour les personnes diabétiques qui souffrent également d’insuffisance rénale nécessitant une dialyse. Cette étude souligne le besoin urgent d’initiatives de recherche et d’amélioration de la qualité pour soutenir ces personnes. Il serait également souhaitable de mener des études qualitatives explorant les perspectives des patients dialysés, des professionnels de la santé et du personnel soignant.

## Introduction

More than 5.7 million Canadians are living with diabetes mellitus (type 1 or type 2) and only half of these individuals have adequate glucose control.^1^ Diabetes is the cause of death for more than 7000 Canadians every year (A Diabetes Strategy for Canada [ADSC], 2019) and is the most common cause of chronic kidney disease (CKD).^1,2^ The CKD affects 13.4% of the population, and approximately half of these cases is caused by diabetes.^3^

Diabetes is a chronic metabolic disorder affecting millions worldwide and is the leading cause of kidney failure globally.^3,4^ Self-management of diabetes is a key component of optimal therapy and requires frequent decisions pertaining to blood glucose control, dietary choices, lifestyle modification, and adherence to medications.^5-7^ Due to a high burden of disease conditions and challenges associated with transitioning to dialysis therapy, the self-management of diabetes becomes challenging for patients with both diabetes and kidney failure requiring dialysis.^8,9^ Altered drug metabolism in this population restricts the use of numerous oral and injectable diabetes medications, while additional dietary constraints, including limitations on potassium and phosphate intake, further add complexity to making healthy food choices.^10^ Although patients with diabetes and kidney failure requiring dialysis are at substantial risk of diabetes-related complications, hospitalizations, and mortality,^11-13^ there is limited literature on diabetes self-management in this group of patients. To promote and enhance self-management practices, it is important to explore and understand the viewpoints of patients as they navigate this challenging time with dual conditions. It is important to identify barriers and facilitators to diabetes self-management to fully support their diabetes care and needs. Thus, the purpose of this narrative review was to gather and synthesize evidence on barriers to, and facilitators of, diabetes self-management from patients with concomitant diabetes and kidney failure requiring dialysis.

## Methods

### Study Design

This study was a narrative literature review. Specifically, empirical integrative review design was utilized.^14,15^ Empirical integrative reviews design analyzes and synthesizes publications of evidence-based studies with diverse methodologies.

### Selection Criteria

Selection criteria for this review were identified using the SPIDER (sample, phenomenon of interest, design, evaluation, and research type) question format.^16^ The *sample* was patients with diabetes and concomitant kidney failure on dialysis; the *phenomenon of interest* was reported or explored as perceived barriers to, and facilitators of, diabetes self-management. Studies of any *design* or *evaluation* with primary *research type* of qualitative, quantitative, or mixed-methods were eligible.

### Search Strategy

A literature search was conducted in CINAHL, EMBASE, and OVID MEDLINE in Fall (2023) with guidance from the Nursing Librarian. Subject headings and keywords used in the search covered 4 concepts: (1) end-stage kidney disease or chronic kidney disease or chronic renal failure; AND (2) diabetes self-management, self-care, or quality of life; AND (3) barriers, or patient-reported barrier or financial barrier or challenges; AND or OR (4) facilitators or enablers (see Table 1) for search strategy in OVID MEDLINE. The search was restricted to English-language articles from the year 1990 to November 2023.

**Table 1.** Search Strategy in OVID MEDLINE.

Number	Searches	Results
1	Exp^a^ Diabetes Mellitus, Type 1/or exp Diabetes Mellitus, Type 2/	243 307
2	Exp multi-morbidity/or co-morbid.mp.	8025
3	Diabetes or diabetic*.mp.	329 530
4	Or/1-3	483 520
5	End-stage renal disease.mp. or exp Kidney Failure, Chronic kidney disease/	39 369
6	(End-stage kidney disease or chronic kidney disease).mp.	81 383
7	5 or 6	113 656
8	Exp health outcomes/	0
9	Exp health-related quality of life/	273 292
10	(Diabetes self-management or self-management or self-management experience).mp.	28 038
11	Or/8-10	297 606
12	(Barrier or challenge or patient-reported barrier or financial barrier).mp	696 705
13	(Facilitator or enabler).mp	10 162
14	4 and 7 and 11 and 13	1
15	4 and 7 and 11 and 12	9
16	Limit 15 to English language	8

a EXP—Explode is used in the literature search to include a term and all its narrower, related terms in the search.

### Study Extraction and Inclusion

The inclusion criteria for review were empirical studies exploring and describing the barriers to, and facilitators of diabetes self-management and overall diabetes management in the context of kidney disease (see Table 2). One hundred thirty-four records were retrieved from all sources; 129 records remained after removing duplicates using *Mendeley* (Reference Manager) software.

**Table 2.** Inclusion and Exclusion Criteria.

Inclusion criteria	Exclusion criteria
Adult patients with both diabetes and kidney disease diagnosis	Studies that included patients with either diabetes or kidney disease
English language	
Original articles	

**Figure 1.**
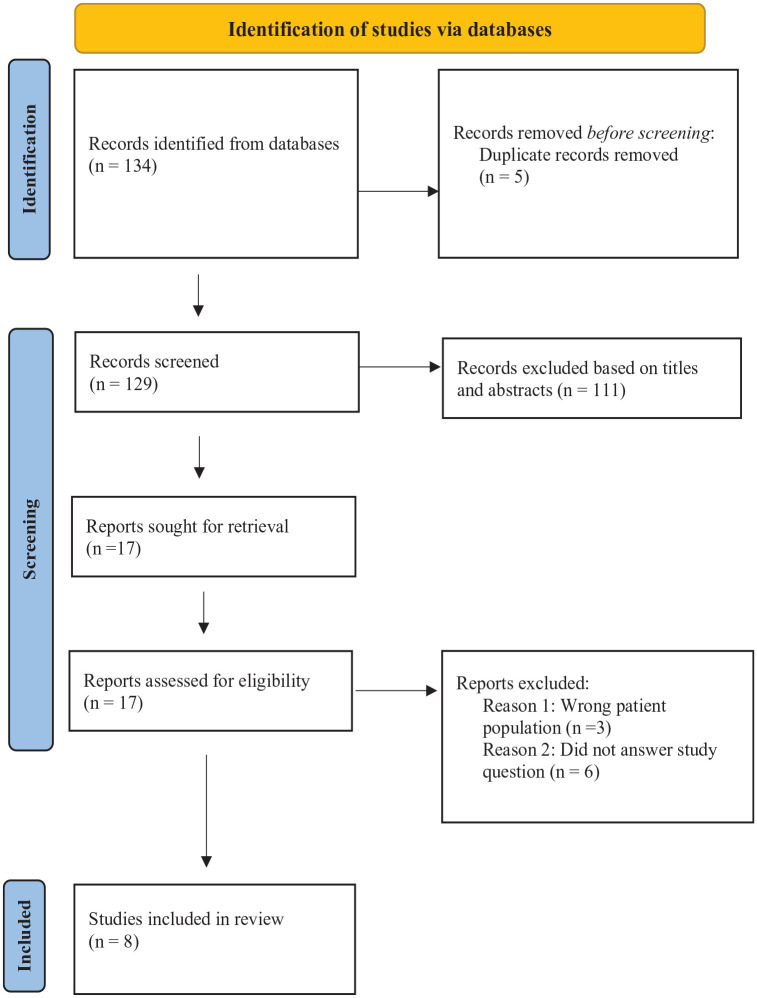
PRISMA flow diagram. (Joanna Briggs Institute, 2023).

After screening titles and abstracts, 17 articles were identified for full-text review (see Figure 1). Two reviewers (K.Y & G.M.) independently screened all titles and abstracts using inclusion and exclusion criteria specified above. Any disagreements about eligibility were resolved through discussion. The reference lists of these 17 articles were then searched to locate additional studies, and none were identified. Studies that did not discuss literature on barriers to, and/or facilitators of diabetes self-management in CKD patients, as well as studies that focused only on kidney disease self-management were excluded. The following information was extracted from each study: country; date; study setting; research design; population/sample size; barriers; facilitators, and other research findings. This information was then compiled to develop a descriptive summary of the literature (see Supplementary Appendix). As new findings were noted in the reviewed literature, additional data points were extracted to aid in robust interpretation and analysis. Thematic analysis, as outlined by Braun and Clarke (2006),^17^ was used to organize, synthesize, and systematically identify the most important themes across multiple studies. Using qualitative data analysis software (NVivo 12), extracted 8 main themes and 16 subthemes (outlined below in Table 3).

**Table 3.** Identified Themes and Subthemes.

Theme	Subtheme	Frequency	Illustrative quotations
*BARRIERS*			
INDIVIDUAL			
Higher burden of health	• High number of medical appointments • Complexity of medical conditions • Prioritization of health issues	Clemens et al^18^ Clemens et al^27^ Lo et al^19^ Zimbudzi et al^20^ Shirazian et al^21^	“They will [providers] set up appointments and then they will end up having appointments at the same time. . . . or sometimes they will set up an appointment for me on this day and then the next one is on the next day, instead of trying to set them up so one is in the morning, and one is in the afternoon.” (Clemens et al^18(p4)^) “Participants also expressed difficulty scheduling appointments with patients and encouraging regular attendance.” (Clemens et al, 2021, p.5) “Some participants were frustrated about the number of doctors they had to see.” (Clemens et al, 2021, p.5) “Patients reporting the presence of other life stressors.” (Shirazian et al^21(p22)^) “Other life stressors unrelated to the patients’ illness, family situation and jobs that made self-care of diabetes and CKD a lower priority.” (Zimbudzi et al^20^, p.4)
Increased complexity of dietary regimen	• Loss of control with cooking • Competing dietary priorities	Clemens et al^18^ Shirazian et al^21^	“With [diabetes] we were taught to eat whole wheat breads and with the renal disease you are taught not to eat any of that, you are taught to eat white, so the two diets counteract. Like they are telling you one thing for sugar, and they are telling you one thing for renal, so yes there is a big change.” (Clemens et al^18(p3)^) “In my house, stuff can show up, and that’s a challenge.” (Shirazian et al^21(p24)^)
COMMUNITY			
Access to care services	• Lack of availability of specialized services • Effect of distance from health care services	Bello et al^22^ Lo et al^19^ Zimbudzi et al^20^	“Remote dwellers were more likely to progress to eGFR < 10 mL/min/1.73 m2 but not initiate RRT.” (Bello et al^22(p4)^)
SYSTEMIC			
Siloed and fragmented care	• Having only 1 health condition addressed • Care fragmentation between multiple services	Clemens et al^18^ Clemens et al^27^ Lo et al^23^ Zimbudzi et al^24^ Shirazian et al^21^	“Say you have a number on one [blood test] and it is out of range, they are like, well, you will have to talk to this specialist about that. We do not deal with that.” (Clemens et al, 2021, p.4) “I keep bringing up, you have gone to computers, why can’t you look this stuff up, all the blood tests, all the results? It is there in front of you, you type in my ID number, and everything comes up. So, why cannot you do that.” (Clemens et al^18(p4)^)
ECONOMIC			
Financial limitations	• Cost of supplies • Difficulties in managing cost of recommended diet	Clemens et al^18^ Zimbudzi et al^20^	“In terms of the cost, a lot of things are not covered. Needles for insulin are not, which I have a bone to pick with that.” (Clemens et al^18(p3)^)
*FACILITATORS*			
INDIVIDUAL			
Self-management support and education	• Taking ownership and responsibility • Better understanding of comorbid conditions • Technology utilization	Clemens et al^18^ Clemens et al^27^ Lo et al^19^	“You should be able to go on a website and see that information, which should be available to you as a patient . . . So that in the age that we now live in, that information is available.” (Clemens et al^18(p4)^) “The best thing I have is an app on my phone . . . It keeps track of all my medications. It keeps track of your vitals, so you can put in your blood glucoses and all that.” (Clemens et al^18(p4)^)
INTERPERSONAL			
Family support	• Instrumental, emotional support	Shirazian et al^21^	“I don’t think I’d be alive truly if it weren’t for my husband.” (Shirazian et al^21(p22)^)
SYSTEMIC			
Coordinated care	• Coordinated, multidisciplinary care	Clemens et al^18^ Lo et al^19^	“Just you go in with one visit and you can cover the gamut. You can talk to the dietitian, and you can talk to wound care. It is all there.” (Clemens et al^18(p4)^)

## Quality Assessment of the Studies

Critical appraisal skills program (CASP) and Joanna Briggs Institute (JBI) tools for qualitative, quantitative, and mixed-methods studies were used to assess the methodological limitations of the included studies. The following domains were considered: validation of study results, research aim, recruitment strategy, exposure and outcome measure, reliability and validation of data collection tools, data analysis, potential for bias, and ethical considerations. Each domain was judged as yes, no, or maybe. Studies were not excluded based on this assessment, in line with the recommendation, but this information was considered in the analysis of the findings, assessment of confidence in the review findings, and the reporting of the review. In general, most of the studies included were of reasonable methodological quality and provided sufficient details on rigor and ethical considerations. Among the 8 studies reviewed, only 3^18,19,21^ utilized qualitative methodology. In addition, only 2 of these studies included patients’ perspectives. These findings underscore the need for further qualitative research to fully understand the depth and breadth of this topic.

## Review

Eight articles examining the concept of diabetes self-management in the context of kidney failure requiring dialysis were included. All articles included in this review were published between the years 2012 and 2021. Five studies were conducted in Australia, 2 in Canada, and 1 in the United States. None of the articles were international in scope; however, 2 studies^20,25^ were conducted across 4 large hospitals in Australia. The articles included qualitative (n = 3), quantitative (n = 4), and mixed-methods (n = 1) approaches.

To extract concepts and categorize barriers and facilitators from the descriptive findings in included studies, a coding process aligned with qualitative methodology was used. Each article and interview transcript was thoroughly reviewed, and key phrases or sentences related to barriers and facilitators of diabetes self-management and overall diabetes management in the context of kidney disease were highlighted. Related codes were grouped together to form broader themes. Finally, connections between themes were mapped by K.Y., who led the primary analysis to illustrate how different barriers or facilitators intersected. G.M. and R.Q. confirmed the findings, while S.R.B. and K.K.S. reviewed and validated the thematic structure to ensure consistency and rigor. The other authors were not involved in the initial coding and mapping process, as this stage was intentionally limited to a smaller group to maintain analytic consistency and reduce interpretive bias.

**Figure 2.**
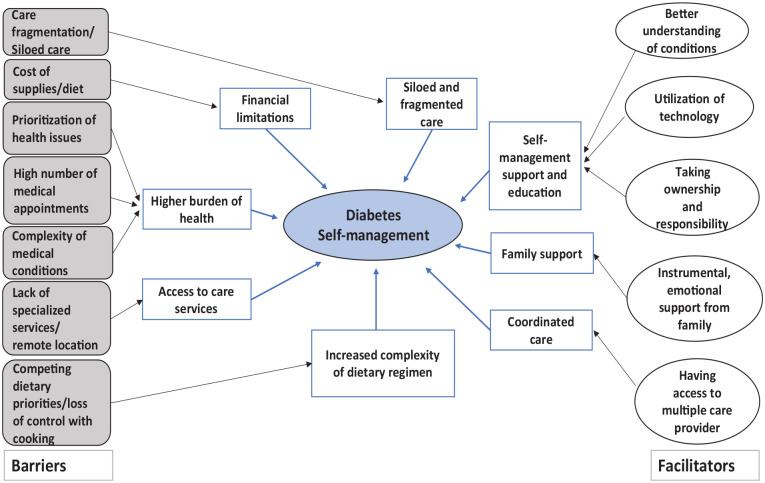
Thematic map illustrating the interrelationships between the themes and subthemes. Download full image: Diabetes Self-management Map.pptx.

The Social-Ecological model developed by Bronfenbrenner^26^ in 1970s was used to categorize the barriers and facilitators. Bronfenbrenner’s Ecological Model was chosen as the guiding theoretical framework for this research because it offers a comprehensive lens for examining human behavior within the context of multiple, interacting environmental systems. This model consists of several interrelated levels of influence on health behaviors and outcomes such as individual, interpersonal, organizational, community, and public policy. Barriers and facilitators were categorized into various levels, which better equipped us to understand not only the range of factors that impact self-management strategies but also the interplay between them. Barriers such as “higher burden of health” and “access to health services” were the most cited perceived barriers, followed by “siloed and fragmented care.” Common facilitators noted included “self-management support and education,” “family support,” and “coordinated care.” Other factors were less frequently reported; however, this does not necessarily reflect lesser importance, as all the included studies were more focused on reporting barriers to self-management of diabetes in the context of kidney failure (see Figure 2 for thematic map). Perspectives were gained from patients, as well as from health care professionals, across the various levels of care with respect to kidney disease.

## Individual Level Barriers

### Higher Burden of Health

“Higher burden of health” was the most frequent barrier identified in the literature. This was particularly pronounced in individuals grappling with both diabetes and kidney disease, a dual burden that elevates the complexity of their medical conditions. This heightened complexity not only places them at an increased risk for hospitalization, morbidity, and mortality^19,21^ but also complicates their daily lives, creating a formidable challenge to manage. The struggle to attend multiple medical appointments becomes a distressing aspect of their daily life, further heightened by the demands of dialysis treatments. Interestingly, the “higher burden of health” is not only an experiential barrier noted by the participants but was also echoed by health care providers. In a study done by Clemens et al,^27^ challenges faced by health care professionals in managing and prioritizing diabetes within the context of multiple, concurrent health issues in this patient population were emphasized.

### Increased Complexity of Dietary Regimen

Although participants understood that a dietary regimen for diabetes self-management was a crucial aspect of managing a chronic disease, they found it difficult to implement recommended changes. Following the diabetic diet is challenging due to the substantial dietary restrictions in the context of kidney failure requiring dialysis. Conflicts between diabetic and renal diets were a commonly reported barrier by participants in 2 studies.^18,21^ Many participants reported diabetic and renal diets to be conflicting as they faced more dietary and fluid restrictions due to kidney failure. Furthermore, they reported having a lack of instructions from experts knowledgeable in both dietary areas.^18,21^ On a similar note, diabetes educators expressed that they had difficulty providing patients with dietary advice, as the recommendations for diabetic and kidney disease diets often conflicted.^27^

## Community

### Access to Care Services

The logistical challenges associated with accessing health care facilities in remote areas can prevent patients from attending medical appointments, or participating in diabetes education programs, leading to gaps in care and poor disease management. A population-based study done by researchers in Alberta, Canada, found that residing in a rural location was a significant barrier to seeking essential medical treatment. Remarkably, remote dwellers in this study were more likely to progress to eGFR <10 mL/min/1.73 m^2^, but not initiate renal replacement therapy, and were more likely to report issues with transportation.^22^ Moreover, the lack of readily available and specialized medical resources in remote areas posed a substantial impediment to the effective delivery of health care,^20^ which contributed to delayed medical attention and may have led to negative health outcomes for these individuals.

## Systemic

### Siloed and Fragmented Care

Participants highlighted the need for better coordination of care, and many felt their care was fragmented in the context of kidney failure.^18,19^ Patients with these multiple comorbidities had complex needs and required providers to address their needs in detail. In a study done by Lo et al,^23^ researchers sought the perspective of general practitioners and tertiary health professionals and reported insufficient levels of integration between various levels of care. Many participants mentioned the pervasive sense of fragmentation in their care experiences, as elucidated in research conducted by Clemens et al^18^ and Lo et al.^19^ The study conducted by Lo et al^19^ delved into the perspectives of both general practitioners and tertiary health professionals, shedding light on the systemic challenge in the integration of care across various levels of the health care continuum, leading to suboptimal experiences for patients and potentially compromising the overall effectiveness of their treatment plans. Effective coordination ensures comprehensive diabetes management, including monitoring blood glucose, diet, exercise, medication adherence, and complications, with regular follow-ups for early issue detection and prompt management.

## Economic

### Financial Limitations

The findings from studies have brought attention to the significant impact of having limited finances on the effective self-management of diabetes. Financial constraints emerged as a notable barrier, particularly in the context of individuals expressing their inability to afford innovative technologies designed to support diabetes management practices, such as continuous glucose monitoring devices.^18,20^ In fact, many participants reported paying out of pocket for supplies, such as insulin needles. The impact of financial barriers on the self-management of chronic disease could be detrimental and affect quality of life for patients with kidney failure. Patients with kidney failure requiring dialysis often face significant financial limitations due to the excessive costs of medical treatments, medications, and transportation, as well as reduced income from decreased work capacity.

## Individual Level Facilitators

### Self-management Support and Education

Participants underscored the importance of receiving comprehensive education from their health care providers regarding the optimal management of diabetes in the context of diabetes and kidney failure with dialysis. Access to accurate and relevant information facilitated patients’ deeper understanding of their health status and strategies to manage care. A survey conducted in Canada shed light on the unique challenges faced by individuals with kidney disease. For example, diabetes educators highlighted the necessity for a specialized approach that prioritizes education and care coordination for this patient population.^27^

## Interpersonal

### Family Support

Participants in numerous studies emphasized the crucial role of self-management support provided by their family members.^18,19^ The steadfast support from loved ones not only served as a source of emotional encouragement but also played a pivotal role in the day-to-day practical aspects of managing their chronic condition. The significant role of family support in fostering effective self-management of diabetes was evident in a study conducted by Shirazian et al.^21^ Participants in this qualitative study highlighted the essential contribution of their family members in navigating the challenges posed by diabetes. A notable aspect of this support system was the collaborative effort to monitor and control food choices by family. Many participants shared instances where family members played an initiative-taking role in fostering a diabetes-friendly environment.

## Systemic

### Care Coordination

Participants in the included studies articulated the advantages associated with the integration of various care services within a single appointment.^18^ Receiving diabetes care within the dialysis unit was particularly emphasized as noteworthy and beneficial by many participants. This simplified the health care process but also proved to be exceptionally convenient for the participants, minimizing the need for multiple appointments and reducing the overall burden of managing their health conditions. Participants in the study by Clemens et al^18^ showed openness to educational sessions and counseling services, as it provided an invaluable opportunity for participants to deepen their understanding of their condition and receive personalized guidance on lifestyle modifications, coping strategies, and adherence to treatment plans.

The literature on barriers to, and facilitators of, diabetes self-management in the context of kidney failure and dialysis has been summarized in this review. Analysis of eligible studies identified barriers to diabetes self-management that covered 5 main themes: increased complexity of dietary regimen, higher burden of health, access to care services, siloed and fragmented care, and financial limitations. Facilitators were grouped into 3 themes: self-management support and education, family support, and coordinated care. Individual- and system-level issues accounted for the bulk of reported barriers, such as a high burden of health, multiple complex health conditions, having a high number of medical appointments and health care providers, in addition to being on dialysis and being diagnosed with diabetes. These findings suggest a need for preventive and integrated care approaches, leveraging patient and community partners’ engagement to mitigate these barriers at various levels. In Australia, an integrated model of care was codesigned and implemented for patients with complex health care needs such as diabetes and kidney failure. The development of this model was guided by input from both patients and health care professionals through focus groups, as well as by semi-structured interviews with caregivers and health care providers. Notable features of this care model include regular screening for psychological well-being, patient support via a dedicated phone advice line, and specialized primary health care assistance in managing conditions such as diabetes and kidney disease. Combined diabetes and kidney services have shown to improve clinical target attainment, such as hemoglobin A_1C_, and may enhance patients’ capacity to self-manage their diabetes.^24,28^ It is widely acknowledged that our health care systems are predominantly structured to address single chronic diseases rather than comorbidities. Nevertheless, with the prolonged life expectancy of individuals managing diabetes, health care teams are increasingly encountering patients with multiple concurrent conditions, underscoring the need for enhanced skills, and coordinated care to effectively meet the diverse needs of clients. The cost of managing diabetes was reported as a barrier by many participants. This prevents individuals from procuring necessary supplies for self-management and highlights the need to address financial barriers. Numerous observational studies have suggested that reducing copayments has the potential to improve adherence.^29,30^ One way to reduce the frequency of financial barriers is to provide full coverage for high-value medications. A multifactorial large-scale randomized controlled trial in Canada recently determined the impact of value-based insurance—a value-based formulary which eliminates copayment for high-value preventive medications—in a broader patient population on both clinical outcomes and costs.^31^ The Assessing Outcomes of Enhanced Chronic Disease Care Through Patient Education and a Value-based Formulary Study was a 2 × 2 factorial randomized trial in Alberta that demonstrated a 22% reduction in adverse clinical events using a tailored self-management education support program.^32^ It showed positive results in terms of adherence to medication regimens for chronic diseases.^29^ There is a compelling need for more interventions at the policy level to study whether financial incentives positively affect self-management. In Canada, health care system is publicly funded, and as such, financial barriers could vary between different health care systems. In terms of facilitators, participants valued self-management education and support from family members. Several studies highlighted the importance of ongoing education and support from health care providers, emphasizing the need for strategies and interventions at the system level to optimize self-management. Electronic and mobile (phone-based) education approaches have been shown to be effective in educating and promoting behavior change in patients with type 2 diabetes.^33-35^ This could be extrapolated to, and explored in, patients with comorbid diabetes and CKD, to allow individual medication dose adjustment, reduce cost and health provider burden, and address barriers around education and self-empowerment. It is important to emphasize efforts to harness facilitators to support patients dealing with both diabetes and kidney failure. All studies included in this review were published within the last 8 years, suggesting an increased interest in comprehending the impact of facilitators on self-management. Multiple factors play a key role in diabetes management; hence, upstream interventions and strategies can be more impactful, as they aim to address social and economic factors, which are the root causes of disease incidence and prevalence.

A notable insight that emerged from this review was the variation in barriers and facilitators to care across different health systems, particularly between countries like Canada, Australia, and the United States. In Canada and Australia, where publicly funded health care systems are more established, common facilitators included better access to community-based support services and integrated care models for coordination of care. In contrast, studies from the United States highlighted significant structural barriers such as fragmented service delivery. These differences in findings underscore the importance of considering health system structures when developing policies or interventions, as facilitators in 1 country may not be transferable to another without accounting for systemic and cultural context.

## Limitations

Our review has limitations. The literature search was in-depth and comprehensive, but not exhaustive, as it is typical of a narrative literature review.^36^ Also, we restricted our search criteria to articles published in the English language; hence, some relevant studies might not have been included. A key limitation of this review is the lack of specification regarding diabetes type and CKD stage in the included studies, which restricted our ability to explore whether barriers and facilitators differed based on disease progression or diabetes classification. This study also has strengths. This review was strengthened by including perspectives of both care providers and patients, as well as the perspective of health professionals to broaden our understanding of the phenomenon. In addition, the literature was critically appraised for the quality and rigor of research methods in the included studies.

## Implications for Future Research

The results of this work demonstrate that implementing tailored interventions to assist patients in managing diabetes could yield beneficial outcomes. Furthermore, the findings from this review underscore the importance of capturing patients’ perspectives and experiences. Although there are extensive qualitative studies on self-management in the general population with diabetes, our review noted a paucity of knowledge concerning self-management of diabetes in individuals with kidney failure on dialysis. The majority of studies included in our review lacked in-depth perspectives from patients due to their quantitative design, highlighting an important gap in the literature. This could have implications for diabetes education, as the personal conceptualization of illness can significantly impact how patients approach their management. Further research is necessary to understand the role of socioeconomic factors in the self-management of diabetes and the complex interplay among these barriers. For example, financial constraints may lead to poor medication adherence, which in turn may contribute to suboptimal glucose control. Conversely, psychosocial factors can affect an individual’s motivation to engage in self-management practices, thereby exacerbating health disparities. Understanding these interactions is vital for developing effective interventions that consider the multifaceted nature of diabetes self-management. Clinicians seeking to implement diabetes care and interventions may want to consider drawing on these strategies.

## Conclusion

While barriers to diabetes self-management are multifaceted and complex, they are not insurmountable, and there are known facilitators to enhance diabetes care and self-management. This narrative review highlights the need for more research in this domain and the importance of using qualitative methodologies to gain insight into patients’ perspectives due to the complex nature of self-management in the presence of multiple chronic health conditions.

## Supplemental Material

sj-docx-1-cjk-10.1177_20543581251359734 – Supplemental material for Barriers to, and Facilitators of, Diabetes Self-management in the Dialysis Population Supplemental material, sj-docx-1-cjk-10.1177_20543581251359734 for Barriers to, and Facilitators of, Diabetes Self-management in the Dialysis Population by Kokab Younis, Graham McCaffrey, Kathryn King Shier, Shelley Raffin Bouchal and Robert R. Quinn in Canadian Journal of Kidney Health and Disease
