# Supplementary material for: Barriers to, and Facilitators of, Diabetes Self-management in the Dialysis Population: A Narrative Review and Implications for Research
Source: Can J Kidney Health Dis. 2025 Jul 29;12:20543581251359734. doi: 10.1177/20543581251359734 (PMC12314261; doi:10.1177/20543581251359734)
Supplement: sj-docx-1-cjk-10.1177_20543581251359734 – Supplemental material for Barriers to, and Facilitators of, Diabetes Self-management in the Dialysis Population [file sj-docx-1-cjk-10.1177_20543581251359734.docx]

**Table 1. Search Strategy in OVID MEDLINE**

| # | Searches | Results |
| --- | --- | --- |
| 1 | Exp Diabetes Mellitus, Type 1/ or exp Diabetes Mellitus, Type 2/ | 243307 |
| 2 | Exp multi-morbidity/ or co-morbid.mp. | 8025 |
| 3 | Diabetes or diabetic*.mp. | 329530 |
| 4 | Or/1-3 | 483520 |
| 5 | End-stage renal disease.mp. or exp Kidney Failure, Chronic kidney disease/ | 39369 |
| 6 | (End-stage kidney disease or chronic kidney disease).mp. | 81383 |
| 7 | 5 or 6 | 113656 |
| 8 | Exp health outcomes/ | 0 |
| 9 | Exp health-related quality of life/ | 273292 |
| 10 | (Diabetes self-management or self-management or self-management experience).mp. | 28038 |
| 11 | Or/8-10 | 297606 |
| 12 | (Barrier or challenge or patient-reported barrier or financial barrier).mp | 696705 |
| 13 | (Facilitator or enabler).mp | 10162 |
| 14 | 4 and 7 and 11 and 13 | 1 |
| 15 | 4 and 7 and 11 and 12 | 9 |
| 16 | Limit 15 to English language | 8 |

**Table 2: Inclusion and Exclusion Criteria**

| **Inclusion Criteria** | **Exclusion Criteria** |
| --- | --- |
| - Adult patients with both diabetes and kidney disease diagnosis. | - Studies that included patients with either diabetes or kidney disease. |
| - English language. |  |
| - Original articles. |  |

**Table 3: Identified Themes and Subthemes**

| **Theme** | **Subtheme** | **Frequency** | **Illustrative Quotations** |
| --- | --- | --- | --- |
| ***BARRIERS*** | | | |
| **INDIVIDUAL** | | | |
| **Higher burden of health** | - High number of medical appointments - Complexity of medical conditions - Prioritization of health issues | Clemens et al., 2019  Clemens et al., 2021  Lo et al., 2016.  Zimbudzi et al., 2018  Shirazian et al., 2015 | - *“They will [providers] set up appointments and then they will end up having appointments at the same time. … or sometimes they will set up an appointment for me on this day and then the next one is on the next day, instead of trying to set them up so one is in the morning, and one is in the afternoon” (Clemens et al., 2019, p.4).* - *“Participants also expressed difﬁculty scheduling appointments with patients and encouraging regular attendance” (Clemens et al., 2021, p.5).* - *“Some participants were frustrated about the number of doctors they had to see” (Clemens et al., 2021, p.5).* - *“Patients reporting the presence of other life stressors” (Shirazian et al., 2015, p.22).* - *“Other life stressors unrelated to the patients’ illness, family situation and jobs that made self-care of diabetes and CKD a lower priority” (Zimbudzi et al., 2018, p.4).* |
| **Increased complexity of dietary regimen** | - Loss of control with cooking - Competing dietary priorities | Clemens et al., 2019  Shirazian et al., 2015 | - *“With [diabetes] we were taught to eat whole wheat breads and with the renal disease you are taught not to eat any of that, you are taught to eat white, so the two diets counteract. Like they are telling you one thing for sugar, and they are telling you one thing for renal, so yes there is a big change” (Clemens et al.,2019, p.3).* - *“In my house, stuﬀ can show up, and that’s a challenge” (Shirazian et al.,2015, p.24).* |
| **COMMUNITY** | | | |
| **Access to care services** | - Lack of availability of specialized services - Effect of distance from health care services | Bello et al., 2012  Lo et al., 2016  Zimbudzi et al., 2018 | - *“Remote dwellers were more likely to progress to eGFR < 10 mL/min/1.73 m2 but not initiate RRT” (Bello et al., 2012, p.4).* |
| **SYSTEMIC** | | | |
| **Siloed and fragmented**  **care** | - Having only one health condition addressed - Care fragmentation between multiple services | Clemens et al., 2019  Clemens et al., 2021  Lo et al., 2017  Zimbudzi et al., 2019  Shirazian et al., 2015 | - *“Say you have a number on one [blood test] and it is out of range, they are like, well, you will have to talk to this specialist about that. We do not deal with that” (Clemens et al., 201, p.4).* - *“I keep bringing up, you have gone to computers, why can’t you look this stuff up, all the blood tests, all the results? It is there in front of you, you type in my ID number, and everything comes up. So, why cannot you do that” (Clemens et al., 2019, p.4).* |
| **ECONOMIC** | | | |
| **Financial limitations** | - Cost of supplies - Difficulties in managing cost of recommended diet | Clemens et al., 2019  Zimbudzi et al., 2018 | - *“In terms of the cost, a lot of things are not covered. Needles for insulin are not, which I have a bone to pick with that” (Clemens et al.,2019, p.3).* |
| ***FACILITATORS*** | | | |
| **INDIVIDUAL** | | | |
| **Self-management support and education** | - Taking ownership and responsibility - Better understanding of co-morbid conditions - Technology utilization | Clemens et al., 2019  Clemens et al., 2021  Lo et al.,2016 | - *“You should be able to go on a website and see that information, which should be available to you as a patient. … So that in the age that we now live in, that information is available” Clemens et al., 2019, p.4)* - *“The best thing I have is an app on my phone. … It keeps track of all my medications. It keeps track of your vitals, so you can put in your blood glucoses and all that” (Clemens et al., 2019, p.4).* |
| **INTERPERSONAL** | | | |
| **Family support** | - Instrumental, emotional support | Shirazian et al., 2015 | - *“I don’t think I’d be alive truly if it weren’t for my husband” (Shirazian et al., 2015, p.22)* |
| **SYSTEMIC** | | | |
| **Coordinated care** | - Coordinated,   multidisciplinary care | Clemens et al., 2019  Lo et al.,  2016 | - *“Just you go in with one visit and you can cover the gamut. You can talk to the dietitian, and you can talk to wound care. It is all there” (Clemens et al., 2019, p.4).* |

**Appendix: Summary of Studies Included in Narrative Review**

| Primary author, Year,  Geography | Aim | Study Design, Sample Size | Barriers | Facilitators |
| --- | --- | --- | --- | --- |
| Clemens et al., 2019  Ontario, Canada | To explore health care experience and solutions for patient-centred diabetes care. | One-to-one interviews, focus groups, 12 participants  (Patients) | Multiple medical appointments and care providers, not enough time with health professionals, care fragmentation and silos, communication gaps, lack of education about health condition, cost of diabetes management, access to technology, and diets | Coordinated care, self-management support, and education |
| Lo et al., 2016  Australia | To explore the perspectives of primary and tertiary care. | Purposive sampling  Semi-structured interviews/focus groups, 65 participants (Healthcare Professionals) | Access to specialty care, lack of coordination of care, lack of preventive approach was identified as key factors that influence healthcare and require improvement.  Poor communication between healthcare providers. | A reactive approach to health, quality improvement initiatives |
| Clemens et al., 2021  Canada | To understand diabetes educators’ experience providing diabetes support to patients with CKD and elicit their view on the additional care needs of this population. | Online Survey Study, 122 participants (Healthcare professionals) | Balancing complex medical conditions, socioeconomic barriers to treatment, language barriers, emotional barriers, extreme hyperglycemia. | Self-management support, coordinated care, medication support |
| Primary author, Year,  Geography | Aim | Study Design, Sample Size | Barriers | Facilitators |
| Bello et al., 2012  Canada | To investigate the relation of residence location, markers of good quality  healthcare and adverse clinical outcomes in patients with diabetes and chronic kidney disease. | Retrospective cohort study,   31,337 participants (Patients) | Remote dwellers with diabetes and kidney disease were less likely to receive appropriate specialist care due to access to care services.  Less likely to undergo the recommended assessment of hemoglobin A1c and proteinuria at the recommended intervals, and less likely to receive appropriate medications due to their geographical location. | Not discussed |
| Lo et al., 2017  Australia | To explore the gaps and barriers in health-care provision for co-morbid diabetes and chronic kidney disease. | Cross-sectional survey, 308 participants (Patients) | Poor continuity of care, inadequate understanding/education about kidney disease, and feeling unwell.  Inadequate support from family and friends, conflicting advice from and poor communication amongst specialists, the effect of co-morbidities on self-management and feeling unmotivated. | Not discussed |
| Zimbudzi et al., 2017  Australia | Patient reported barriers to health care and low physical and mental well-being among people with diabetes and CKD. | Survey study, 308 participants  (Patients) | Impact of the disease on family and friends,  feeling unwell, having other life stressors that make self-care a low priority, unavailability of home help, low mood, low self-efficacy, and psychosocial barriers. | Not discussed |
| Primary author, Year,  Geography | Aim | Study Design,  Sample Size | Barriers | Facilitators |
| Lo et al., 2019  Australia | To explore the association between patient-reported barriers to healthcare, patient activation, quality of life and diabetes self-care. | Cross-sectional study, 199  Participants  (Patients) | Receiving conflicting advice from specialists, poor continuity of care, inadequate understanding and education about kidney disease and trouble maintaining dietary and fluid restrictions. | Not discussed |
| Shirazian et al., 2023  United States | The self- management experience of patients with Type 2 diabetes and chronic kidney disease. | Qualitative study, 23 Participants  (Patients) | Emotional reaction to dialysis, high burden of health. | Family support |
